# Assimilates mobilization, stable canopy temperature and expression of expansin stabilizes grain weight in wheat cultivar LOK-1 under different soil moisture conditions

**DOI:** 10.1186/s40529-017-0169-7

**Published:** 2017-03-21

**Authors:** Mahesh Kumar, Susheel Kumar Raina, Venkadasamy Govindasamy, Ajay Kumar Singh, Ram Lal Choudhary, Jagadish Rane, Paramjit Singh Minhas

**Affiliations:** 1 School of Drought Stress Management, ICAR-National Institute of Abiotic Stress Management (NIASM), Malegaon, Baramati, Pune, 413 115 India; 2 ICAR-Central Institute of Temperate Horticulture, Old Air Field, PO Rangreth, Srinagar, J & K 190007 India; 3 Division of Microbiology, ICAR-Indian Agricultural Research Institute, New Delhi, 110012 India; 4 School of Edaphic Stress Management, ICAR-National Institute of Abiotic Stress Management, Malegaon, Baramati, 413 115 India; 5 ICAR-National Institute of Abiotic Stress Management, Malegaon, Baramati, 413 115 India

**Keywords:** Wheat, LOK-1, Source-sink relations, Grain growth, Grain weight, Canopy temperature

## Abstract

**Background:**

Grain yield of wheat is primarily determined by both grain number and grain weight, which often influence each other in response to environmental stimuli. Some of the genotypes are capable of maintaining high single grain weight (SGW) across the environments. Understanding mechanisms and factors associated with the superiority of such genotypes over others is necessary to enhance productivity of wheat.

**Results:**

Experiments were conducted to elucidate the physiological basis of high SGW of LOK-1, a wheat cultivar grown in dry and hot environments in the central and peninsular zones of India. SGW of LOK-1 was least affected by removal of spikelets indicating little competition between the grains within the spike for assimilates. Reduction in SGW due to defoliation was less and the contribution of stem reserves to the grain development was high in LOK-1 relative to other cultivars. It seems that high level of expression of genes such as expansin *(TaExpA6)* contributes to the high SGW of LOK-1.

**Conclusions:**

Source was not a limiting factor for grain growth of LOK-1 in contrast to other cultivars, whereas sink appeared to be a limiting factor in recently released/identified cultivars. Differences in the amounts of water soluble stem carbohydrate reserves translocated to grain could be one of the factors contributing to higher grain weight in LOK-1. High level expression of *TaExpA6*, one of the genes contributing to the elongation of endosperm, seems to be crucial for grain growth in wheat.

**Electronic supplementary material:**

The online version of this article (doi:10.1186/s40529-017-0169-7) contains supplementary material, which is available to authorized users.

## Background

To meet the food demand of the world population projected to reach over 9 billion by 2050, production of wheat (*Triticum aestivum* L.) needs to be increased by 70–100% (Godfray et al. 2010). This has to be accomplished with available natural resources including the land vulnerable to abiotic stresses caused by supra-optimal ambient temperature and soil moisture deficit. Since the predicted climate change can amplify the frequency and magnitude of these stresses, enhanced efforts are needed for genetic improvement of stress tolerance in temperate crops like wheat. The key components of wheat grain yield viz., grain number and grain weight are highly vulnerable to high temperatures and soil moisture deficit (Dreccer et al. 2009; Vignjevic et al. 2015). However, some genotypes of wheat can maintain high SGW across the varying environments (Mohammad et al. 2012). Several attempts have been made to elucidate the basis of variation in yield components, particularly the size of the grain (Lopes et al. 2012; Bustos et al. 2013; Rebetzke et al. 2013; Aisawi et al. 2015). Often, the seed weight has been associated with both source and sinks limitations (Reynolds et al. 2007; Álvaro et al. 2008). This can also be influenced by differences in assimilates supply from current photosynthesis and pre-anthesis assimilates stored in the stem (Blum 1998; Rane et al. 2003; Fischer 2011; Vignjevic et al. 2015). Other traits such as cooler canopy also facilitates better grain development and hence the productivity of wheat (Mason and Singh 2014).

Recently, genes contributing to SGW have been demonstrated in crops like rice, barley and wheat (Ashikari et al. 2005; Weng et al. 2008; Zalewski et al. 2010; Zhang et al. 2012). Starch is the major storage reserve of wheat grains. Grain weight is primarily determined by the photo synthetic productivity, assimilation and translocation of starch in the developing grain during the crop growth (Jenner et al. 1991). In higher plants, chlorophyll is the major pigment contributing to photosynthesis which is pre-requisite for starch biosynthesis. The stability and net content of chlorophyll can be modulated by cytokinin, a phytohormone (Chang et al. 2015). *TaCKX6*-*D1*, a rice orthologue of *OsCKX2* (*O. sativa Cytokinin oxidase 2*), has been associated with grain weight in wheat (Zhang et al. 2012). Specific suppression of *OsCKX2* enhanced tiller number and grain weight in transgenic rice (Yeh et al. 2015). Amylopectin, the more abundant polymer of starch is synthesized by the coordinated actions of AGPase, soluble starch synthase (SS), starch branching enzyme (BE) and starch debranching enzyme (DBE) (Kang et al. 2013). Other genes coding for expansins have also been implicated in wheat grain development. Lizana et al. (2010) reported abundance of *TaExpA6* transcripts in the pericarp and endosperm of wheat at 187 thermal time (°Cd) (10 days after anthesis) close to the peak expression detected by RT-PCR. All these reports prompted us to investigate the role of genes like *TaCKX6*-*D1*, expansin and starch synthase in contributing to higher grain growth in LOK-1 cultivar of wheat. However, many of the hypotheses related to grain weight cannot be generalized due to genotype by environment interaction and location specific trait evaluation.

LOK-1, one of the popular cultivars of wheat, in India for the last 20 years owing to its superior quality grains. This cultivar yields stable and large grains across the environment though the yield potential is relatively less than the recently released cultivars for the region (AICW & BIP Report 2012–13).

We conducted the experiments to elucidate the physiological and molecular basis of differences between SGW of LOK-1 and that of other recently released cultivars. The main objective was to identify factors responsible for high SGW in the former genotype by assessing contribution of source, sink, current photosynthesis assimilate, pre-anthesis stem reserves, canopy temperature and genes associated with the grain development in wheat.

## Methods

### Growing conditions and plant materials

Field experiments were conducted at ICAR-NIASM experimental farm, Baramati, Maharashtra located in western part of India (18°9′N, 74°28′E) during three growing seasons, 2012–2013, 2013–2014, and 2014–2015. The experimental site was featured by 60–70 cm deep black soil with about 30% silt and 40% clay layered over native basaltic soil. Each experiment consisted of a split plot design with four replications and plots of 2 by 1.5 m (4 or 6 rows 0.23 m apart). Locally adapted high yielding cultivars namely NIAW-34, NIAW-301, HD-2189 and LOK-1 were chosen for the studies (Table 1; Additional file 1: Table S1). Experiments were executed between 10–20th of November and 30th of March in all cases. Planting was carried out manually with a seed rate of 100 kg ha^−1^. Mean temperature during grain filling was around 25.5 °C during the 3 years, with average maximum temperature of 29 °C. Seasonal precipitations ranged from 8 to 76 mm. In addition, flood-irrigation with 250 mm of water was provided during each crop season to ensure sufficient soil moisture. There were no rains during the grain development phase and the post anthesis water stress was imposed by withholding irrigation a week before anthesis. Experimental plots were fertilized before planting with 60 kg Nitrogen, 60 kg P_2_O_5_ and 40 kg K_2_O ha^−1^. Additional nitrogen of 30 kg ha^−1^ was applied at crown root initiation and at maximum tillering stage represented by Zadoks scale 1.3 and 2.8 respectively (Zadoks et al. 1974). Weeds were controlled manually throughout the experimental period.

**Table 1:** Genetic, physiological and yield characteristics of four wheat cultivars used in the study

Cultivar name	Pedigree	Year of release	Days to anthesis^a^	Days to physiological maturity^a^	Number of tillers/m^2^		Yield (g/m^2^)	
					Well water	Water stress	Well water	Water stress
LOK-1	S-308 × S-331 (Sonalika × Chhotilarma)	1979	57–65	81–86	335	289	426	396
HD-2189	HD 1963/HD 1931	1980	68–72	86–102	348	261	454	405
NIAW-34	CIANO-79/(SIB)Parula	1997	62–70	88–108	309	273	394	368
NIAW-301	Seri-82/Hork	2002	65–70	90–105	325	286	404	370
CD (p = 0.05)		Between stress levels at same variety			21.9		24.95	
		Between varieties at same stress level			24.15		27.59	

^a^Based on experimental data

### Measurements and analyses

#### Soil moisture measurements

**Fig. 1:**
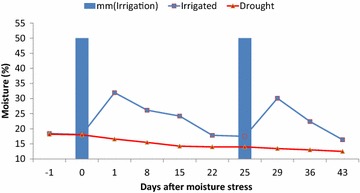
Soil moisture status in well water and water stress plots during wheat grain filling stage in 2014–15 crop seasons

Soil samples from each experimental unit were collected in aluminum boxes with secure lids every week at two depths (15 and 30 cm) by using augers. The samples were weighed immediately and then oven dried at 105 °C for 72 h for determining soil moisture content by gravimetric method (Fig. 1).

#### Grain growth

At anthesis, 65–70 identical main shoot spikes of each cultivar from every experimental units were ear marked. Five spikes were harvested to measure grain dry weight at every 3rd or 4th day beginning from day of anthesis till 30 days after anthesis (physiological maturity). The harvested spikes were dried in an oven at 70 °C for 72 h. Grains were separated from the middle six spikelets of each of the five spikes for measuring SGW (Laxman et al. 2014). Grain dry weight (mg) of each cultivar plotted against growing degree days after anthesis (GDDAA).

#### Sink limitation

At 50% anthesis, four pairs of identical main-stem spikes (a total of 8) were selected and tagged in each plot (Ma et al. 1996). Since the spikes in each pair had the same number of spikelets, alternate spikelets from one of the spikes were removed by hand to reduce the sink size by 50% while other spike was kept as a control. It was assumed that the removal of half of the spikelets will double the assimilate supply to the remaining spikelets on the spikes if source is a limiting factor. At maturity, the ears were harvested separately and grains were separated from the spike manually for recording the number of spikelets, grains per spike and grain weight. SGW was measured by dividing the grain weight per spike at approximately 12% moisture level by the number of grains per spike.

#### Source limitation

Seven days after anthesis, four pairs of main stem spikes (a total of 8 ears) were selected and tagged in each plot (Ma et al. 1996). In each plant only main stem was retained and rests of the tillers were removed to ensure the uniformity in sink and source during this experiment. Leaves of one of the main stem in each pair were completely removed to prevent the supply of current photosynthesis assimilates for the developing grains, while identical main stem of neighboring plant in each plot was kept as control. At harvest, all the pairs of main stems were collected and grains were separated by hand for measuring grain number and grain weight.

#### Net CO_2_ assimilation rate

Net CO_2_ assimilation rate was recorded through photosynthesis system (GFS 3000; Walz, Germany) during 10.00 AM to 2.00 PM in the fully expanded flag leaf (Biswas et al. 2013). Data were recorded three times in the flag leaf at 55, 71 and 88 DAS. Reference CO_2_ levels were maintained at 400 µmol s^−1^, and chamber flow was set at 750 µmol s^−1^. Ambient photosynthetically active radiation (PAR) during the measurements ranged from 1000 to 1200 µmol m^−2^ s^−1^, while ambient humidity ranged between 30 and 40%.

#### Canopy temperature

Canopy temperature was recorded by thermal imager (VarioCam^®^hr inspect 575, Jenoptic, Germany) that operates in the wave bands of 8–14 µm with a thermal resolution of 0.01 °C. Thermal images with spatial resolution of 768 × 576 pixels were captured seven times covering crop growth stages just before and after anthesis. A tripod perpendicular to the area being imaged was used to ensure constant distance and angle between the camera and crop canopy for during all the measurements. Dry and wet references were used to mimic leaves with fully closed and fully opened stomata, respectively (Jones et al. 2002) and to avoid extreme conditions while capturing images. Emissivity for all the measurements were set at 0.96 (Jones 2004). IRBIS^®^ software (Jenoptic, Germany) was used to analyse thermal images. A total of six areas of interest in each thermal image were outlined manually for analysis. Images in the visible wavelength were referred to exclude noise from ground area. Minimum canopy temperature in each of the sections in each thermal image was considered to get a value that represents cooling capacity of cultivar. Since the variation among the cultivars for this trait at any given point of time was marginal, we opted to compute area under curve of canopy temperature vs days with reference to anthesis for assessing genetic variation in plant’s capacity to keep the canopy cooler.

### Estimation of stem water soluble carbohydrates (WSC)

Five plants at anthesis and at harvest were randomly selected and separated into leaf blades, and stems along with leaf sheath. All samples were dried at 70 °C for 48 h and weighed. The WSC in the stems was measured at both anthesis and maturity. WSC were extracted from 0.1 g of ground stem material by extracting once with 8 mL of 80% ethanol at 80 °C in water bath followed by two extractions with 8 mL distilled water at 70 °C. Each extraction was carried out for at least 1 h. The extract was centrifuged at room temperature for 10 min at 4000*g* and the extracts were combined. Total soluble sugars in the samples were analyzed by the anthrone method (Yemm and Willis 1954). Mobilization efficiency of assimilates was estimated by the proportion (%) of WSC at physiological maturity to post anthesis for maximum WSC (Ehdaie et al. 2006).

### qRT-PCR analysis of genes associated with grain development

Developing grains of all the four cultivars were collected 8 days after anthesis to assess the expression of *TaCKX6*-*D1*, *TaExpA6* and *TaSSIIA* genes. For each variety, three biological samples, each represented by the mixed samples at the same phonological growth stage from at least three individual spikes, were used for qRT-PCR. Three independent amplifications were performed on each biological sample. Total RNA was extracted with RNA extraction kit (Qiagen, Germany). DNA was removed by digestion with DNAse I (Thermo Scientific, USA) before reverse transcription. The RNA samples were quantified using a UV–Visible spectrophotometer (SMA-3000) and 2 µg of total RNA were used for first-strand cDNA synthesis using Superscript cDNA synthesis kit (Invitrogen, USA). Allele-specific primer pairs for *TaCKX6*-*D1* (Zhang et al. 2012) and *TaExpA6* (Lizana et al. 2010) were used to detect transcripts of *TaCKX6*-*D1* and *TaExpA6* although primers for starch synthase IIA (*TaSSIIA*) were designed with the help of Primer3 plus software using the sequence available in NCBI. Amplification of glyceraldehyde-3-phosphate dehydrogenase (*GAPDH*) was used as an internal control for relative quantification. qRT-PCR was performed on a Biorad make CFX96 machine using the iQ SYBR Green master mix (Biorad, USA). The procedure used was initial polymerase activation for 30 s at 95 °C followed by 40 cycles of 95 °C for 10 s and 60 °C for 30 s. Data from the quantitative analysis of expression were transformed by the 2^−ΔΔCt^ method (Livak and Schmittgen 2001). The details of primers used in the study are given in Table 2.

**Table 2:** Details of the primers used in the study

Primer name	Primer sequence	Product size (bp)	References
C19EF4	5′-CGACGAGATCTTACGGTTCT-3′	~230	Zhang et al. (2012)
C19ER4	5′-GACCGATGGATCAGCCA-3′		
GAPDHF	5′-TTAGACTTGCGAAGCCAGCA-3′	~81	This study
GAPDHR	5′-AAATGCCCTTGAGGTTTCCC-3′		
TaExp6F	5′-CAATCCTCCCCGCGA AC-3′	~217	Lizana et al. (2010)
TaExp6-R	5′-GGTCCCCTTCACCGACAT-3′		
TaSSIIAF	5′- ACGTCATGAACGTGGTCGTC-3′	~198	This study
TaSSIIAR	5′-CCTGTCCAGCAGCCTTGTA-3′		

### Experimental design and statistical analysis

The field experiment was conducted in split plot design in four replications. However, individual plants were considered as replications except for canopy temperatures and net CO_2_ assimilation rate. Data were analysed by two way ANOVA technique using the SPSS statistical software (version 16) to determine the effect of each treatment. If the F-ratio was significant, a multiple mean comparison was performed using Duncan Multiple range Test (DMRT) at α-0.05. Simple statistics with analysis of variance were also computed to differentiate the treatment effects by using statistical analysis software package SAS^®^ 9.3.

## Results

### Weather during crop season

**Fig. 2:**
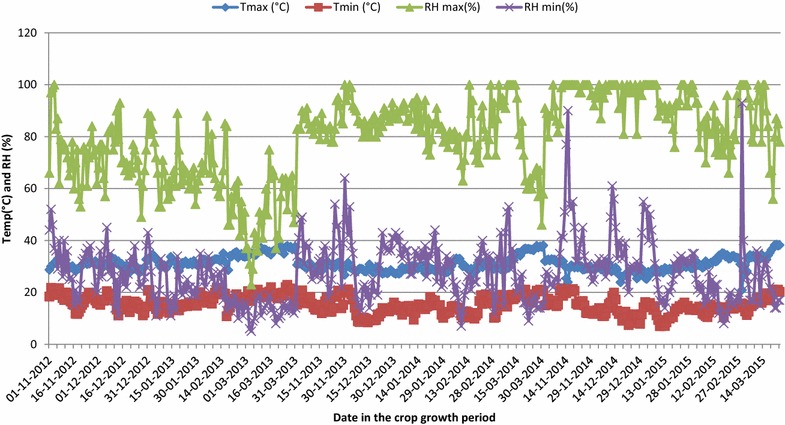
Temperature (max and min) Relative humidity (max and min) of the day during three crop seasons for the period from 2012–15

During the crop season, average minimum temperatures ranged from 14.3 to 17.0 °C while the average maximum temperature ranged from 30.5 to 32.3 °C (Table 3; Fig. 2). Crop season 2012–13 was the hottest and 2014–15 was the coolest among all three crop season throughout the crop growth and more so at grain development.

### Changes in unit grain weight across grain growth period

**Fig. 3:**
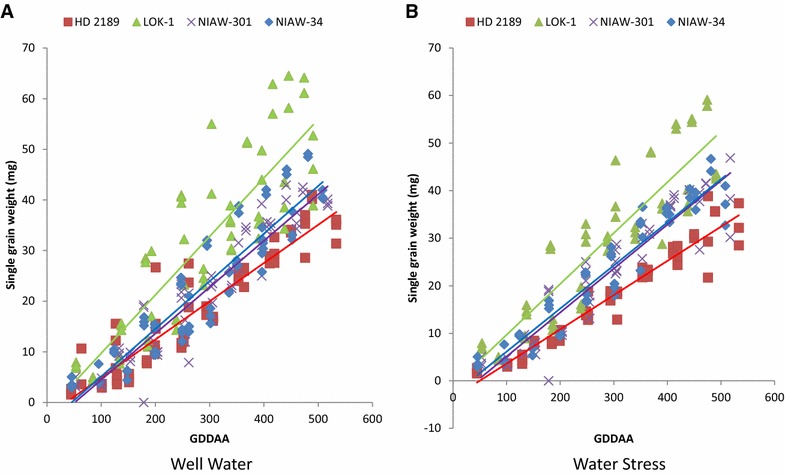
Changes in grain dry weight of 4 wheat cultivars with GDDAA during three crop seasons (2012–2015) under well watered (**A**) and water stress condition (**B**). Observation was recorded at 3–4 days interval after anthesis (each point represent average of 6–8 grain from each 10–12 spikes)

Results clearly revealed that grains of LOK-1 accumulated more photosynthesis/starch assimilates than other cultivars under both well watered and water stress conditions in the field during grain growth (Fig. 3). The difference between SGW of LOK-1 and other variety was conspicuous during all three crop seasons. Rate of grain growth as represented by slope of trend line plotted for grain weight vs growing degree days after anthesis further revealed that LOK-1 accumulates assimilates faster than other cultivars.

### Effect of spikelet removal on SGW

**Fig. 4:**
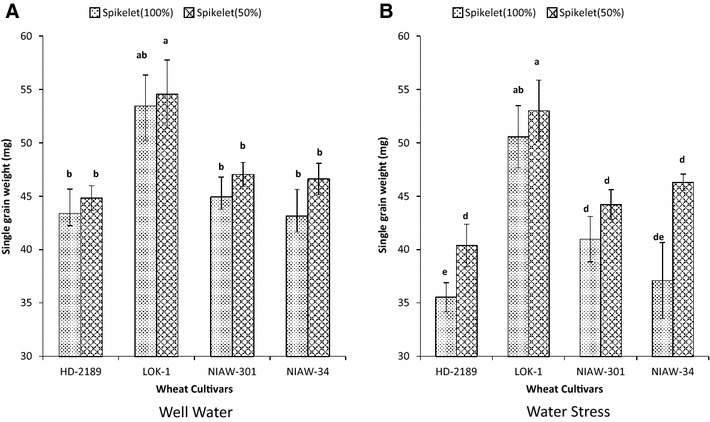
Genetic variation in grain weight at harvest with 100 or 50% grains per spike on main stem during grain growth under well watered (**A**) and post anthesis moisture stress (**B**) in four wheat cultivars. (n = 8). Means with *same letter* are not significantly different at p < 0.05

Reduction in average number of grains per spike as a result of removal of spikelets ranged from 20.5 to 31.4 across the genotypes and soil moisture levels. Consequently, increase in SGW in response to spikelet removal ranged from 4.8 to 24.8% under soil moisture stress and 2.1–8.0% under ample soil moisture conditions (Fig. 4A). However, there was no significant impact of removal of spikelets on SGW of LOK-1 under both well water and water stress condition. In contrast, there was significant increase in the SGW of HD-2189 and NIAW- 34 particularly under soil moisture deficit (Fig. 4B).

SGW of LOK-1 was not sensitive to variation in grain number caused by removal of grains in contrast to other tested cultivars (Additional file 2). This suggested that more grain weight in LOK-1 was not necessarily due to less number of grain, but as a result of intrinsic sink capacity to accommodate the assimilates relatively in larger quantity as compared to other genotypes.

### Effect of defoliation on SGW

**Fig. 5:**
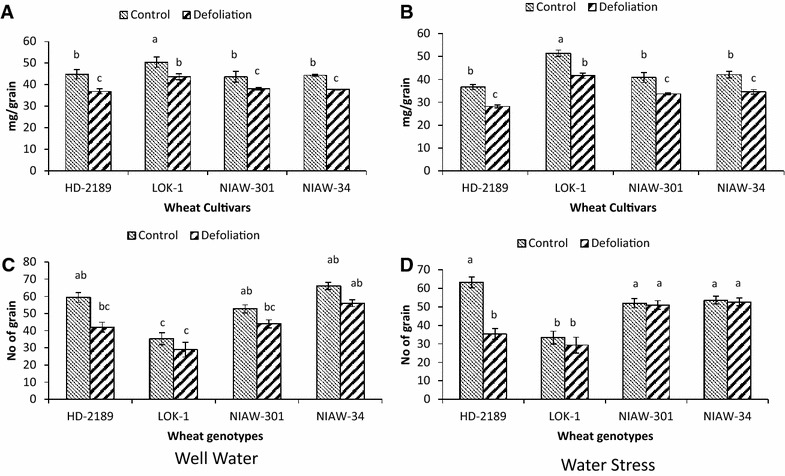
Genetic variation in single grain weight (**A**, **B**) and grain number (**C**, **D**) in presence and absence of leaves on main stem during grain growth under well water (**A**, **C**) and post anthesis water stress (**B**, **D**) in four wheat cultivars. Defoliation was done 7 days after anthesis. Means with *same letter* are not significantly different at p < 0.05

Defoliation caused a reduction in SGW to an extent of 12.5–17.6% in all the cultivars under well water conditions, while the reduction was 17.4–23.0% under limited moisture conditions (Fig. 5A, B). LOK-1 and NIAW-301 exhibited marginal reduction in grain weight compared to others indicating that these cultivars utilize reserve carbohydrates for grain development and leaves as source was not a limiting factor.

Impact of defoliation was also noticed on number of grains per spike, with the largest reduction in HD-2189 as compared to others (Fig. 5C, D). However, defoliation mediated reduction in grain number under both moisture conditions in other cultivars was not significant.

### Effect of soil moisture on Net CO_2_ assimilation rate (P_n_)

**Fig. 6:**
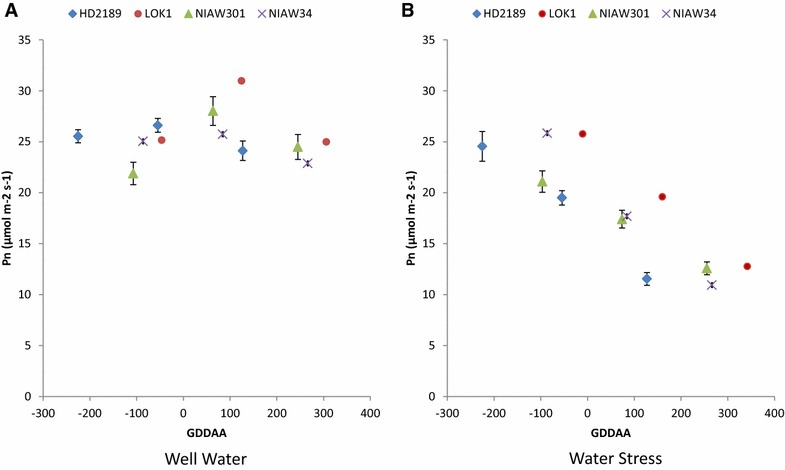
Relationship between net CO_2_ assimilation rate and thermal time under well water (**A**) and water stress (**B**) conditions in four cultivars. Each point is mean of 6-8 data point

Mean net CO_2_ assimilation rate ranged from 24.3 to 28.0 and 14.2–16.2 µmol m^−2^ s^−1^ under well water and deficit soil moisture conditions respectively (Fig. 6A, B). In general under water stress net CO_2_ assimilation rate in decreasing trend. Under well water conditions LOK-1 had higher Pn after anthesis (Fig. 6A) as compared to other cultivars.

### Effect of soil moisture on canopy temperature

**Fig. 7:**
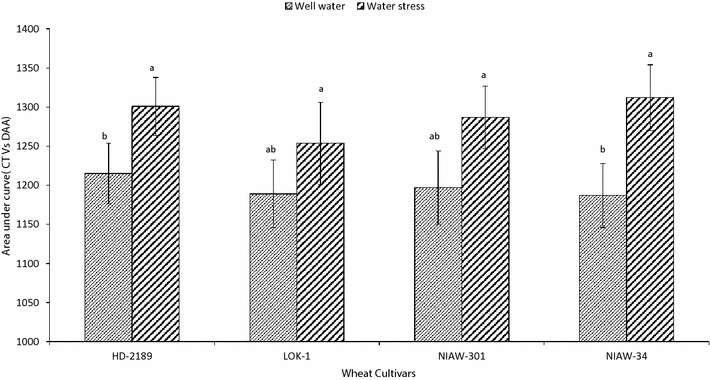
Genetic variation in canopy temperature (min) under well watered and post anthesis water stress in four wheat cultivars. Area under curve was estimated from the graph of canopy temperature vs days After Anthesis. Means with *same letter* are not significantly different at p < 0.05

Canopy temperature for all the four cultivars was recorded throughout the growing period in well watered as well as water stress conditions. Area under curve plotted with minimum temperature observed in the canopy vs days after anthesis (DAA) indicated that canopy temperature did not vary across the genotypes (Fig. 7). However, soil moisture deficit did not significantly affect the canopy temperature of the cultivar LOK-1 and NIAW-301. In other two cultivar canopy temperature is higher under water stress plots.

### Effect of soil moisture on mobilization efficiency

**Fig. 8:**
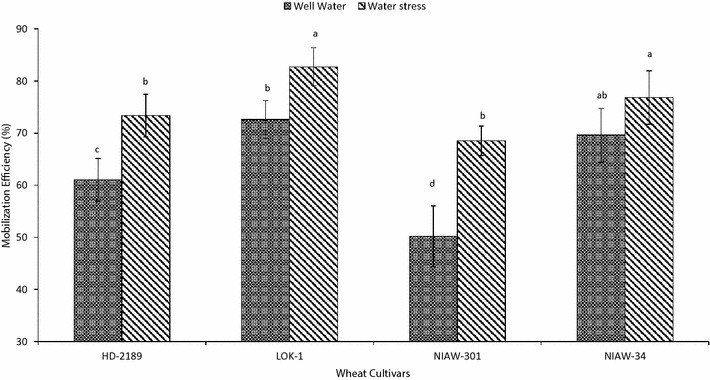
Mobilization efficiency (%) of stem WSC in four wheat cultivars under well watered and soil moisture stress conditions. Means with same letter are not significantly different at p < 0.05

Mobilization of WSC from the stem to economic part was found in the range of 50.2–72.6% in the well water conditions and 68.5–82.7% under soil moisture limited conditions after anthesis. WSC mobilization in LOK-1 was up to 44.6 and 20.7%, in NIAW-34 was up to 38.6 and 12.15% higher than the same in other cultivars in the presence and absence of post anthesis soil moisture stress respectively (Fig. 8).

### Expression analysis of genes associated with the grain development

**Fig. 9:**
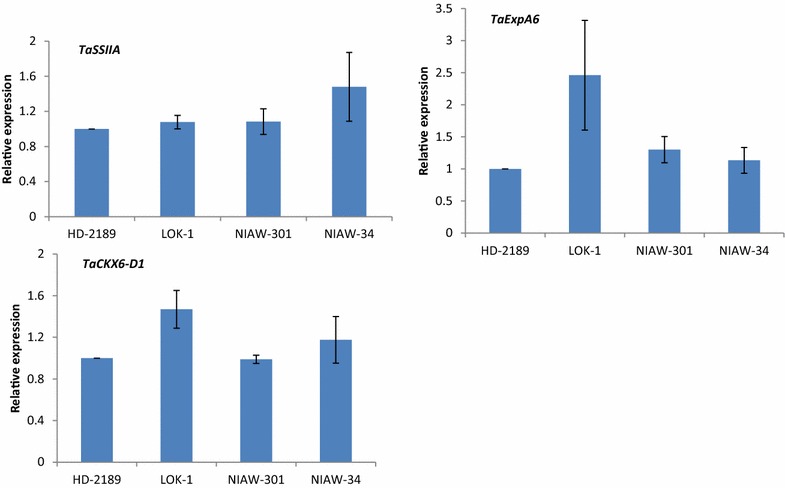
Quantitative RT-PCR analysis of *TaCKX*-*D1*, *TaEXpA6* and *TaSSIIA* genes in HD-2189, LOK-1, NIAW-301 and NIAW-34 wheat genotypes. Expression levels were normalized against expression of *T. aestivum* GAPDH gene as an internal control and are shown relative to HD-2189 as control genotype. The relative level of each gene in control genotype was standardized as 1. Values are presented as the mean and the *errors bars* indicate standard errors of triplicate samples

The wheat cultivar, LOK-1 exhibited maximum accumulation of *TaCKX6*-*D1* transcripts with an approx. 1.5-fold increase compared to HD-2189, NIAW-301 (Fig. 9). *TaExpA6* transcripts accumulated approx. 2.5-fold in LOK-1 compared to HD-2189. However, maximum *TaSSIIA* transcript accumulation was recorded in NIAW-34 followed by NIAW-301 and LOK-1 revealing almost identical levels (Additional file 1: Table S2).

## Discussion

Several studies have shown that the higher the temperature the lighter the weight of grains at maturity (Sofield et al. 1977; Chowdhury and Wardlaw 1978; Stone and Nicolas 1994; Wardlaw 1994; Savin and Nicolas 1996; Rane et al. 2007). In addition, soil moisture stress also severely affects the development of grain weight in wheat under different environmental conditions (Konopka et al. 2007). However, despite adverse environmental conditions some wheat genotypes maintain their grain weight. For our investigations, we choose bold grained cultivar LOK-1, one of the mega cultivars of wheat being grown in hot and dry conditions prevailing in Central and Peninsular part of India for more than two decades although it has now turned susceptible to diseases. Though its grain yield potential is less, grain weight of LOK-1 is relatively higher than those of other recently developed cultivars even under hot and dry environment. Since the grain weight and its stability are critical determinants of final grain yield we probed possible reasons for higher and stable grain weight of LOK-1 relative to three other cultivars, which are popular in these regions.

### Weather conditions during the crop seasons on SGW and total grain yield

Ambient temperatures during 2012–13 crop seasons were higher by about 2 °C than the consecutive 2 years. In addition, the humidity was substantially low and there were negligible rains during the first year (Table 3). In contrast, there were no much differences between 2013–14 and 2014–15 with respect to weather parameters. Consequently, the grain weight in general was lower during the first year relative to the same recorded during the next consecutive years. The warmer climate is affecting growth and development of plant and grain filling (Porter and Moot 1998). Irrespective of weather, the LOK-1 had higher single grain weight than other cultivars both under water stressed and well watered condition. Rate of grain filling was faster in LOK-1 in the first year as compared to other years under both water stressed and well watered conditions (Fig. 3a, b). Differences among varieties in SGW in the response of moisture stress are thought to occur because moisture stress tolerant varieties maintain faster rates of grain growth temperature-sensitive varieties, at high temperatures (Wheeler et al. 1996).

**Table 3:** Different weather parameters during three wheat crop growing seasons for the period of 2012–2015

Weather parameters	2012–2013	2013–2014	2014–2015
Maximum temperature (°C)	32.3	30.4	30.5
Minimum temperature (°C)	17.0	15.0	14.3
RH maximum (%)	65.2	83.9	91.6
RH minimum (%)	23.3	29.6	29.8
Rain (mm)	8.7	76.2	74.1

### Higher SGW was not due to fewer grains per spike

The two major yield components viz., grain number and SGW are determined at different times of the growing season and therefore are subjected to different conditions and stresses. While success of floret set at pre-anthesis largely determines grain number (Miralles et al. 1998; Gonzalez et al. 2003), SGW is dependent on the extent to which post-anthesis conditions favor grain-fill. However, grain number and weight are interrelated and these results in a capacity to compensate to a certain extent for loss in grain number by increase in SGW and many combinations of these two components are possible (Peltonen-Sainio et al. 2007). With increase in grain number SGW decreases concomitantly in wheat, but independently of any competitive relationship between growing grains (Acreche and Slafer 2006). Under well water conditions LOK-1 maintained higher SGW than other cultivars during all the three crop seasons. Even post anthesis water stress did not affect the grain filling of LOK-1 when compared with other cultivars. This signifies the recalcitrance of SGW trait of LOK-1 to environmental stress.

Increase in SGW in response to degraining can range from 0.5 to 13.2% (Aisawi et al. 2015). Degraining increased SGW by 3.3–8% in recently released cultivars such as HD-2189 and NIAW-34 but it was ineffective in LOK-1. Our attempt to manipulate sink size by partial grain removal revealed that LOK-1 maintained a higher SGW than that of other cultivars in all the range of grain numbers per spike indicating absence of compensation mechanisms involving these two critical traits.

### Current photosynthesis and pre-anthesis assimilates supply for grain development

Grain filling in small-grain cereals occur by acquisition of assimilates from both the current photosynthesis and through translocation of stored non-structural assimilates accumulated before anthesis. In environments featured by gradual increase in temperature and decrease in soil moisture, current photosynthesis during grain filling declines and the remobilization of stored non-structural carbohydrates becomes particularly important for grain filling (Blum 1998). Previous studies on durum wheat have shown that total dry matter translocated from the main stem to the developing grains reach values of up to 25% of the main stem biomass translocated (Álvaro et al. 2008), which are lower than those found in modern bread wheat cultivars (Ehdaie et al. 2006). Stem reserves can contribute 20–40% of the final grain weight in non-stressed environments (Van Herwaarden et al. 1998; Dreccer et al. 2009; Vignjevic et al. 2015) and this can be up to 70% under stressed conditions during grain filling (Tahir and Nakata 2005; Ehdaie et al. 2006; Rebetzke et al. 2008). Hence, we measured current photosynthesis and grain weight in the absence or presence of active leaves during grain growth to explain difference in SGW among the cultivars. There was difference in net rate of CO_2_ assimilation in flag leaves between LOK-1 and other cultivars during grain development even under post-anthesis water stress. This was apparent particularly during the peak phase of assimilates accumulation in grains indicating that this might be one of the reasons for higher single grain weight. In addition, important contribution of pre-anthesis reserves was also evident in LOK-1 from both defoliation and WSC experiments. Further, in contrast to other cultivars, LOK-1 had constant SGW even after reduction in sink size resulted from removal of spikelets at anthesis, which suggested that there was no competition for assimilates among the grains within the spike. It is largely accepted that stem reserve mobilization in grains is affected by sink size, environment and variety. Mobilization efficiency and sink strength (number of grains per spike and grain weight) are two component traits involved in the extent of contribution of stored reserves to grain yield in wheat (Ehdaie et al. 2006). NIAW-34 with higher grain number per spike and LOK-1 with higher SGW explains similar mobilization efficiency in both the cultivar.

### Impact of canopy temperature on SGW

Capacity to keep the canopy cool has been often associated with grain yield in wheat (Mason and Singh 2014). Canopy temperature has been shown to correlate with physiological traits contributing to grain yield under field conditions (Reynolds et al. 2007; Lopes and Reynolds 2010; Rebetzke et al. 2013). We hypothesized that the higher SGW in LOK-1 could be due to its capacity to keep its canopy cooler for longer duration during grain development. Instead of routine method of associating grain yield with canopy temperature measured at any given point of time during crop growth, we considered duration of cooler canopy as represented by area under curve of canopy temperature vs days after anthesis. Here we found that genotype tested in present experiment have similar canopy temperature dynamics during their growth period. However in LOK-1 behave almost similar in both moisture stress as well as normal soil moisture conditions.

### Genes associated with the grain growth and Development

In order to get an insight into molecular mechanism governing grain growth in wheat, we studied the transcript abundance of *TaCKX6*-*D1*, *TaExpA6* and *TaSSIIA* genes in the four genotypes used in present studies. Since minimum grain growth rate was recorded in HD-2189, it was considered as control genotype for comparing the relative transcript abundance of different genes. Cytokinin oxidases/dehydrogenases (CKX) catalyze the irreversible degradation of the CKs iso-pentenyl adenine, zeatin and their ribosides in a single enzymatic step by oxidative side-chain cleavage (Schmülling et al. Schmu¨lling et al. 2003). Gene coding for this enzyme has been found to play a role in determining the grain development in rice (Ashikari et al. 2005), barley (Zalewski et al. 2010) and wheat (Hess et al. 2002; Zhang et al. 2012). Hence, an attempt was made in this study to elucidate the role if any of these genes in determining SGW of wheat. Previously, maximum accumulation of cytokinin oxidase/dehydrogenase (CKX) transcripts was reported 8 days after pollination in wheat. When we measured transcripts abundance for *TaCKX 6*-*D1* gene 8 days after anthesis in four wheat cultivars, maximum expression was observed in LOK-1 indicating possibility of diminishing CKs activity in this cultivar due to increased inhibition of cytokinin catabolism. This is in consistency with earlier report that CKs gradually decrease after the soft-dough stage (Hess et al. 2002). However, further investigations are needed to probe deletions, if any in the sequences of the gene or factors that bring changes at post translational levels which might be contributing to enhanced grain size in wheat.

Cell growth in plant is primarily constrained by cell wall which must be loosened to allow for cell expansion and expansin gene play a role in the cell wall loosening (Cosgrove 2005). Since expansins have been associated with the grain size in wheat (Lizana et al. 2010), we quantified the expression of *TaExpA6*, one of the genes contributing to the elongation of endosperm, which is crucial for grain growth. The possible role of this gene in determining the size of the grain was evident from approx. 2.5 fold enhanced expression in LOK-1 compared to HD-2189.

Starch synthase genes are known to regulate biosynthesis of amylose as well as elongation of glucan/glucose side chains in amylopectin during starch synthesis in the grains of wheat. Wheat grains exposed to elevated temperatures exhibited reduced grain growth and decreased activity of soluble starch synthase in grains (Prakash et al. 2009). In the present study, maximum expression of *TaSSIIA* was recorded in NIAW-34, although there was no appreciable change in its levels across the other genotypes tested- NIAW-301, LOK-1 and HD-2189, indication of a probably minor role for this gene in the development of grain size.

## Conclusions

- Our experiment confirmed that LOK-1, a wheat cultivar known for bold seeded grain, accumulates more biomass as compared to recently released cultivars for the hot and dry regions of India, both under well water and soil moisture stress conditions.
- Source was not a limiting factor for grain growth of LOK-1 in contrast to other cultivars.
- Sink appeared to be a limiting factor in recently released high yielding wheat cultivars like NIAW-34 but not in LOK-1.
- The difference in single grain weights between LOK-1 and any of the other varieties is not due to differences in canopy temperature; however, this genotype has greater capacity to maintain its canopy temperature cooler even under soil moisture deficit.
- Differences in the amounts of water soluble stem carbohydrate reserves could be one of the factors contributing to higher single grain weight in LOK-1.
- *TaExpA6*, one of the expansin genes contributing to the elongation of endosperm, seems crucial for grain growth.

## Additional files

**Additional file 1: Table S1.** Tiller/ m^2^ and yield/m^2^ of four wheat cultivar under well watered and water stress conditions. **Table S2.** Mean efficiency corrected Cq- values of different genes in wheat genotypes.

**Additional file 2.** Final SGW (at physiological maturity) of four wheat genotypes at varying range of grains retained on each spike at the time of anthesis. Error bars derived from 6 to 8 observations. Means with same letter are not significantly different at p<0.05.

## Abbreviations

SGW: single grain weight
GDDAA: growing degree days after anthesis
WSC: water soluble carbohydrates
DAS: days after sowing
