# Supplementary material for: Assimilates mobilization, stable canopy temperature and expression of expansin stabilizes grain weight in wheat cultivar LOK-1 under different soil moisture conditions
Source: Bot Stud. 2017 Mar 21;58:14. doi: 10.1186/s40529-017-0169-7 (PMC5432918; doi:10.1186/s40529-017-0169-7)
Supplement: Supplementary file 1 — Additional file 1: Table S1. Tiller/ m2 and yield/m2 of four wheat cultivar under well watered and water stress conditions. Table S2. Mean efficiency corrected Cq- values of different genes in wheat genotypes. [file 40529_2017_169_MOESM1_ESM.docx]

**Table** **S1: Tiller/ m2 and yield/m2 of four wheat cultivar under well watered and water stress conditions**

|  | **Tiller/m^2^** | | | | | **Yield (g/m^2^ )** | | | | |
| --- | --- | --- | --- | --- | --- | --- | --- | --- | --- | --- |
| **Stress** | **HD2189** | **LOK-1** | **NIAW301** | **NIAW34** | **Mean** | **HD2189** | **LOK-1** | **NIAW301** | **NIAW34** | **Mean** |
| well watered | 348 | 335 | 325 | 309 | 329.25 | 454 | 426 | 404 | 394 | 419.5 |
| water stress | 261 | 289 | 286 | 273 | 277.25 | 405 | 396 | 370 | 368 | 384.75 |
|  |  |  |  | **SEm±** | **CD (P=0.05)** |  |  |  | **SEm±** | **CD (P=0.05)** |
| Between stress levels at same variety | | | | 7.29 | 21.9 | Between stress levels at same variety | | | 8.47 | 24.95 |
| Between varieties at same stress level | | | | 8.36 | 24.15 | Between varieties at same stress level | | | 9.44 | 27.59 |
| Mean | 304 | 312 | 305 | 291 | 303 | 429 | 411 | 387 | 381 | 402 |

**Table S2: Mean efficiency corrected Cq- values of different genes in wheat genotypes**

| Gene* | Genotype | Biological replicate1 | Biological replicate 2 | Biological replicate 3 |
| --- | --- | --- | --- | --- |
| *C19E* | HD-2189 | 24.47 | 24.05 | 25.23 |
| *C19E* | LOK-1 | 24.26 | 24.17 | 24.99 |
| *C19E* | NIAW-301 | 24 | 24.13 | 25.24 |
| *C19E* | NIAW-34 | 25.61 | 23.52 | 24.69 |
| *Exp6* | HD-2189 | 26.03 | 26.83 | 27.29 |
| *Exp6* | LOK-1 | 26.39 | 25.89 | 26.09 |
| *Exp6* | NIAW-301 | 25.73 | 26.36 | 26.57 |
| *Exp6* | NIAW-34 | 27.17 | 26.25 | 26.93 |
| *SSIIA* | HD-2189 | 20.3 | 19.88 | 20.43 |
| *SSIIA* | LOK-1 | 20.3 | 20.36 | 20.92 |
| *SSIIA* | NIAW-301 | 19.5 | 20.09 | 20.34 |
| *SSIIA* | NIAW-34 | 21.53 | 18.83 | 19.57 |
| *GAPDH* | HD-2189 | 17.82 | 16.99 | 17.64 |
| *GAPDH* | LOK-1 | 17.77 | 17.76 | 18.18 |
| *GAPDH* | NIAW-301 | 17.44 | 16.96 | 17.61 |
| *GAPDH* | NIAW-34 | 18.52 | 16.86 | 17.65 |

**C19E : TaCKX6; Exp6: TaExpA6; SSIIA: TaSSIIA; GAPDH: TaGAPH*
