# Supplementary material for: Assimilates mobilization, stable canopy temperature and expression of expansin stabilizes grain weight in wheat cultivar LOK-1 under different soil moisture conditions
Source: Bot Stud. 2017 Mar 21;58:14. doi: 10.1186/s40529-017-0169-7 (PMC5432918; doi:10.1186/s40529-017-0169-7)
Supplement: Supplementary file 2 — Additional file 2. Final SGW (at physiological maturity) of four wheat genotypes at varying range of grains retained on each spike at the time of anthesis. Error bars derived from 6 to 8 observations. Means with same letter are not significantly different at p<0.05. [file 40529_2017_169_MOESM2_ESM.pptx]

## Slide 1
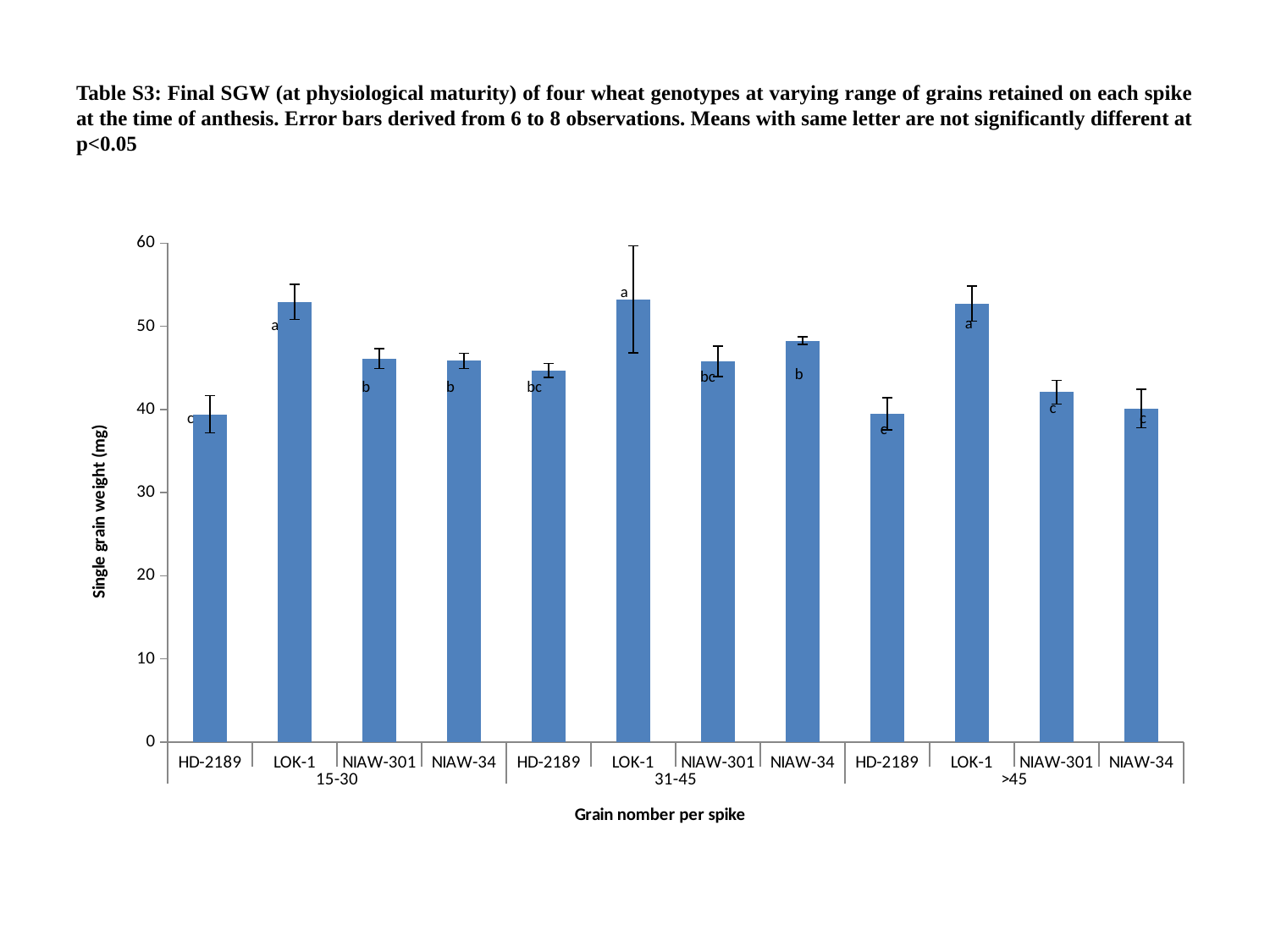

# Table S3: Final SGW (at physiological maturity) of four wheat genotypes at varying range of grains retained on each spike at the time of anthesis. Error bars derived from 6 to 8 observations. Means with same letter are not significantly different at p<0.05
### Chart
| Category | |
|---|---|
| HD-2189 | 39.426023351960374 |
| LOK-1 | 52.94104763944557 |
| NIAW-301 | 46.11608388927229 |
| NIAW-34 | 45.85115258304913 |
| HD-2189 | 44.6949484767025 |
| LOK-1 | 53.24896608767577 |
| NIAW-301 | 45.77501780626781 |
| NIAW-34 | 48.28299120234603 |
| HD-2189 | 39.476152837910114 |
| LOK-1 | 52.752077278623055 |
| NIAW-301 | 42.098011778570736 |
| NIAW-34 | 40.13056210650552 |a
a
a
b
bc
b
b
bc
c
c
c
c
